# Interventions to improve executive functioning and working memory in school-aged children with AD(H)D: a randomised controlled trial and stepped-care approach

**DOI:** 10.1186/1471-244X-13-23

**Published:** 2013-01-11

**Authors:** Marthe LA van der Donk, Anne-Claire Hiemstra-Beernink, Ariane C Tjeenk-Kalff, Aryan V van der Leij, Ramón JL Lindauer

**Affiliations:** 1 Department of Child and Adolescent Psychiatry, Academic Medical Centre Amsterdam, University of Amsterdam, Meibergdreef 5, 1105 AZ, Amsterdam, the Netherlands; 2 De Bascule: Academic Center for Child and Adolescent Psychiatry, PO Box 303, 1115 ZG, Duivendrecht, the Netherlands; 3 Research Institute Child Development and Education, Roeterseiland - Building G, University of Amsterdam, Nieuwe Prinsengracht 130, 1018 VZ, Amsterdam, the Netherlands

**Keywords:** AD(H)D, School-aged children, Working memory training, Executive function training, Academic performance, Randomised controlled trial

## Abstract

**Background:**

Deficits in executive functioning are of great significance in attention-deficit/hyperactivity disorder (ADHD). One of these executive functions, working memory, plays an important role in academic performance and is often seen as the core deficit of this disorder. There are indications that working memory problems and academic performance can be improved by school-oriented interventions but this has not yet been studied systematically. In this study we will determine the short- and long-term effects of a working memory - and an executive function training applied in a school situation for children with AD(H)D, taking individual characteristics, the level of impairment and costs (stepped-care approach) into account.

**Methods/design:**

The study consists of two parts: the first part is a randomised controlled trial with school-aged children (8–12 yrs) with AD(H)D. Two groups (each n = 50) will be randomly assigned to a well studied computerized working memory training ‘Cogmed’, or to the ‘Paying attention in class’ intervention which is an experimental school-based executive function training. Children will be selected from regular -and special education primary schools in the region of Amsterdam, the Netherlands. The second part of the study will determine which specific characteristics are related to non-response of the ‘Paying attention in class’ intervention. School-aged children (8–12 yrs) with AD(H)D will follow the experimental school-based executive function training ‘Paying attention in class’ (n = 175). Academic performance and neurocognitive functioning (primary outcomes) are assessed before, directly after and 6 months after training. Secondary outcome measures are: behaviour in class, behaviour problems and quality of life.

**Discussion:**

So far, there is limited but promising evidence that working memory – and other executive function interventions can improve academic performance. Little is know about the applicability and generalization effects of these interventions in a classroom situation. This study will contribute to this lack of information, especially information related to real classroom and academic situations. By taking into account the costs of both interventions, level of impairment and individual characteristics of the child (stepped-care approach) we will be able to address treatment more adequately for each individual in the future. Trial registration: Nederlands Trial Register NTR3415.

## Background

Studies show that children with Attention Deficit Hyperactivity Disorder (ADHD) often suffer from deficits in executive functions, such as attentional control, inhibition and working memory [1,2]. Stimulant medication may reduce the behaviour symptoms of ADHD but they can also have serious side effects and the effects on cognitive- and executive functioning remain unclear [3]. It is known that these executive functions play an important role in academic performances [4]. Especially working memory skills are associated with problems in learning and poor classroom behaviour [5,6]. Working memory refers to the function of actively holding in mind and manipulating information relevant to a goal [7]. Individuals with poor working memory functioning are at risk of poor educational progress, over 85% of children with poor working memory have problems in reading or mathematics [8]. Recently, studies have shown that training of the working memory has positive xeffects for children with ADHD, and/or learning disabilities [9,10]. Children who followed the computer-based working memory training program (Cogmed Working Memory Training, 10) not only showed improved working memory abilities over time, but also showed better performance on related executive function tasks such as logic reasoning and response inhibition and still continued to show effects after 3 months [9,10]. Interestingly, Holmes and colleagues [11] examined the effects of this working memory computer-based training on measures of academic ability. Adaptive working memory training proved to be associated with higher achievement on mathematic and working memory tasks immediately and after a period of 6 months. In this study a classroom analogue of a working memory task (e.g. following instructions) was carried out, which showed improvement as well. So far, research aimed to study the effects of working memory training on academic performance is promising [11,12]. However, few studies have paid attention to the transfer effects of working memory training on behaviour in a classroom setting. Recently, Green and colleagues [13] showed that working memory training in children with ADHD leads to significant reductions of ‘off-task’ behaviour during academic task performance. But they also noted that the absence of measurable effects of working memory training on teacher ratings in previous studies is a notable limitation of the training. Also, methodological issues such as information bias using only teacher reports and having a small sample size [12] were limitations in earlier studies. The aim of our first study is to replicate the above results of working memory training in a Dutch population of school-aged children with AD(H)D. Moreover it will add specific information on academic performance and behaviour in a classroom setting. From a clinically point of view it is expected that improved working memory also has positive effects on functioning in daily life, therefore we will also focus on quality of life. Besides replicating and extending the results from earlier Cogmed studies, we also compare Cogmed with an experimental school-based executive function training. We developed this training from a clinical point of view. Cogmed is a licensed intervention which creates significant costs for healthcare institutions, healthcare insurance companies and parents. Also, from clinical practice we know that (special aid) teachers lack knowledge about executive functioning and its relation to academic performance and behaviour in class. For instance, they need more specific information and practical tools to guide these impaired children throughout their school career, as described by Gathercole and Alloway [8]. Cogmed is a core working memory training, not contextual to a school environment, and teachers are not involved in the treatment when following the Cogmed protocol. Our ‘Paying attention in class’ intervention is an executive function training, contextual to the school environment. This will be extensively explained below in the Method section.

### Aims

(1) The aim of the first study is to examine the effects of a working memory – and executive function training on academic performance, neurocognitive functioning, behaviour in class, behaviour problems and quality of life of children with AD(H)D between the age of 8 and 12 years.

(2) The aim of the second study is to investigate which specific characteristics are related to non-response of the ‘Paying attention in class’ intervention. By offering Cogmed to the non-responders, we will obtain insight into the effectiveness of both interventions, taking into account individual characteristics, the level of impairment and the costs (stepped-care approach).

With the result of both studies we hope to obtain more insight in the effective elements of both interventions so that treatment can be more individually tailored in the future.

## Methods/design

### Approval

The ethics approval was obtained from the Medical Ethical Committee (2011_269) at the Academic Medical Centre in Amsterdam, the Netherlands.

### Design

The first study is a randomised controlled trial in which children will be randomly assigned to the Cogmed RM [10] intervention (n=50) or the experimental ‘Paying attention in class’ intervention (n=50). We expect that for some children treatment can not be started immediately after enrollment. They will be placed on a waiting list. An extra measurement of academic performance and neurocognitive functioning (primary outcomes) will be administered for the children on the waiting list, this will be a control group (n=50). The second study partially replicates and also extends the first study. This second study investigates which specific characteristics are related to a non-response of the ‘Paying attention in class’ intervention, which requires 175 children including the 50 children from the first study. Based on ethical considerations, Cogmed RM [10] will be offered to the non-responders of the ‘Paying attention in class’ intervention after the follow up assessment (6 months).

### Setting

We will approach three types of primary schools in the region of Amsterdam, the Netherlands: regular primary, special primary education and special education schools. The research team closely collaborates with expert divisions on Special Education and ADHD of the Bascule, Academic Centre for Child and Adolescent Psychiatry, Amsterdam.

### Recruitment

For this study children will be recruited in two ways. First, children from clinical care can be enrolled by contacting a member of the research team. Second, children can be enrolled by their school. In both cases, recruitment will take place by approaching a principal and/or remedial teacher via email or telephone. A special information meeting will be held to extensively inform the staff and ask for permission to participate in the study. Once a school agrees to participate, we will work with a staff member (usually remedial teacher or school psychologist) to select children that meet the criteria for participation. Then parents of these selected children will be approached and informed by the school staff member, followed by an extra information meeting especially for parents. Parents receive an application package including an informed consent form. Training will be started after the informed consent is signed by both parents.

### Inclusion and exclusion criteria

#### Inclusion

Children participating in this study are between the age of 8 and 12 years and diagnosed with AD(H)D by a professional according to the guidelines of the Diagnostic and Statistic Manual of Mental Disorders DSM-IV [14]. Children with co morbid Learning Disabilities (LD) and/or Oppositional Defiant Disorder (ODD) are included as well. Both children with or without medication (e.g. stimulants) are included. Children with medication are only included when they are well adjusted to their medication, which means they are not participating in a medication trial and type and dosage of medication is unchanged at least 4 weeks prior to the start and during the training.

#### Exclusion

Exclusion criteria are (a) psychiatric diagnoses other than AD(H)D/LD/ODD; (b) Total Intelligence quotient <80; (c) significant problems in the use of the Dutch language; (d) severe sensory disabilities (hearing/ vision problems).

### Randomisation

For the first study, the Clinical Research Unit of the Academic Medical Centre composed a randomisation list, stratified by age. Children will be individually randomised to either the Cogmed RM [10] or ‘Paying attention in class’ intervention group by a researcher independent of the research team. This researcher will subsequently inform the therapist about the allocated condition for each child. The PhD student and research assistant will be blind for the allocation.

### Coaches

Developmental psychologists will be trained as ‘training aides’ according to the Cogmed working memory training protocol. They will also be trained as therapists for the ‘Paying attention in class’ intervention. This training consists of an interactive 3 hour course, provided by a member of the research team. For both interventions the developmental psychologists will receive weekly supervision by a certified Cogmed Coach and clinical staff member of the Bascule in which they can discuss the progress and clinical difficulties.

All supervision sessions will be registered with the aid of a structured format. Also, the trainers will fill out a logbook per child daily, for observations and special circumstances. Finally the Cogmed Training Web and the ‘Paying attention in class’ workbook will be used to monitor the results of the training. These three documents will be used to create a checklist for evaluating treatment fidelity.

### Interventions

#### Cogmed working memory training RM

Cogmed Working Memory Training RM is specifically used for children between the age of 7 and 17 years. It consists of a variety of computerized, game-format, working memory tasks that are adaptive, which means that difficulty level is being adjusted automatically to match the working memory span of the child on each task. The program includes 12 visuospatial and verbal working memory tasks, eight of these tasks (90 trials in total) are being completed every day [10]. The child follows the standard Cogmed RM protocol which means following the computer training program for 5 weeks, 5 times a week, approximately 45 minutes a day. The program is provided via the internet on a laptop and the child is trained individually at school, guided by a trained developmental psychologist. Cogmed is a licensed intervention that entails significant costs for healthcare professionals / institutions, health care insurance companies and (depending on type of health care insurance) also parents. The Cogmed Coach Training costs €950, exclusive of a monthly license fee which is dependent on the amount of licenses used. A user ID for one child costs €99. In some cases the monthly fee and user ID costs are reimbursed by the healthcare insurance but there always is a statutory contribution of €100 (in the Netherlands), which is often paid by parents.

#### Paying attention in class

‘Paying attention in class’ is an experimental executive function training that has been developed by our research team. The child is trained individually for 5 weeks, 5 times a week, approximately 45 minutes a day; the same duration as in the Cogmed protocol. This ‘Paying attention in class’ has three aims. First of all, the most important aim of this intervention is to offer psycho education about executive functions that are related to classroom behaviour. By making children more aware of these executive functions needed for adequate classroom behaviours, they obtain more insight in their own learning behaviour. The psycho education addresses five executive functions that are important in a learning situation, namely: paying attention, planning skills, working memory, goal-directed behaviour and metacognition. It will be offered through a MP3 player, based on a ‘brain castle’ theme in which specific compensatory strategies and pitfalls are explained to the child. Every session starts with psycho education that is related to one of the above mentioned executive functions. After hearing this, the child is asked to make a neuropsychological - and school task related exercise in which the psycho education has to be applied. The second aim of this intervention is to train working memory functions. This is done by three adaptive working memory tasks: one visual-, one verbal-, and one instruction paradigm task (30 trials in total). The child will be encouraged to use the compensatory memory-strategies from the psycho education. The third and final aim of this intervention is to make a transfer to a classroom-situation, this will be achieved through several ways. First of all, the strategies introduced through the psycho education as described above will be illustrated and practiced by performing school related tasks. Second, the child brings a registration card to class that reminds him/her of the practiced skill of the day (for example, ‘I repeat what is said’). This card includes the name of the specific compensatory strategy. By using a registration card, the child will be reminded what to practice in the classroom. Also it will inform the teacher about the specific executive function and strategy that is practiced on that day so that he/she can reward the child for applying it in class. Finally, to optimize the transfer from the training session to a situation in class we closely involve the teacher in the process by informing him/her with the protocol and by giving him/her an active part in the process. The teacher will receive psycho education about the use of executive functions in the classroom through a written manual, which will increase their knowledge how to recognize and stimulate such behaviour in learning situations. The teacher also receives structured observation forms daily in which he/she can record whether the child applies the practiced strategies in class. The ‘Paying attention in class’ intervention is experimental, so the exact costs are unknown, but we are aiming at a price of €25 a workbook. The costs of a ‘Paying attention in class’ training will be equal to that of the Cogmed Coach Training but there will not be a monthly license fee.

### Measures and procedures

After written informed consent is obtained, parents will fill out an application package which contains questionnaires of demographic and background information such as previous care and medication use. The measurements of academic performance and neurocognitive functioning (primary outcomes) are administered in a child face-to-face meeting which takes place at three consecutive moments: at baseline, directly after treatment and 6 months after treatment. The test battery that will measure academic performance consists of an automated math [15], a spelling skill test [16] and technical literacy [17] test. The neurocognitive functioning test battery consists of tests that measure attention [18], verbal [19,20] and spatial working memory [21], inhibition [20], planning and goal-directed behaviour [22]. Also, both parents and teachers fill out the ‘The Behaviour Rating of Executive Functions’ [23] questionnaire. The secondary outcome measures of this study are behaviour in class, behaviour problems and quality of life of the children. This will also be measured at the three consecutive moments mentioned above. Behaviour in class will be reported by the teacher using the ‘Leervoorwaardentest’, a questionnaire [24]. Both teacher and parents will rate behaviour problems using ‘The Child Behavior Checklist for Ages 6–18 [25] and ‘Teacher’s Report Form for Ages 6-18’ [26]. Before training is started, the Diagnostic Interview Schedule for Children [27] and Social Communication Questionnaire [28] is administered to parents to rule out other behavioural problems or psychiatric problems that meet exclusion criteria. Finally, quality of life is measured by using the Kidscreen-27 [29] questionnaire and is completed by parents and the child. All tests and questionnaires have been selected based on results from a small pilot study and clinical experience. Most of the tests and questionnaires are in line with other relevant international literature. Other aspects that were taken into account were duration of administration, variety of neurocognitive tests that can establish possible near and far transfer effects and methodological points such as presence of Dutch norms and test-retest reliability. Table 1 gives an overview of all the assessments and outcome measures.

**Table 1:** Outcomes measures

	**Measure**	**Informant**	T1	T2	T3
*Primary outcomes*	*Creature counting:* subtest of Test of Everyday Attention for Children [18], measures attentional control and switching	child	*	*	*
	*Score!:* subtest of Test of Everyday Attention for Children [18], measures sustained attention	child	*	*	*
	*Digit span:* subtest of Wechsler Intelligence Scale for Children-III [19], measures verbal working memory	child	*	*	*
	*Span board:* subtest of Wechsler Non Verbal [21], measures visual working memory	child	*	*	*
	*Word list interference:* subtest from Nepsy-II-nl [20], this subtest is designed to asses verbal working memory, repetition and word recall following interference.	child	*	*	*
	*Six part test:* subtest from Behavioural Assessment of the Dysexecutive Syndrome for Children [22], measures planning, task scheduling and performance monitoring	child	*	*	*
	*Inhibition:* subtest from nepsy-II-nl [20], measures the ability to inhibit automatic responses in favor of novel responses and the ability to switch between respons types.	child	*	*	*
	*Behaviour Rating of Executive Functions (BRIEF):* 70 item questionnaire designed to assess executive functioning in the home and school environments [23]	parent/teacher	*	*	*
	*Dictation (PI dictee,* 16): measures spelling skills	child	*	*	*
	*Automated math (Tempo Test Automatiseren,*15): measures degree of automation of addition, subtraction, multiplication and division.	child	*	*	*
	*Speed reading (Een Minuut Test,* 17): measures technical literacy	child	*	*	*
	*Comprehension of instruction:* subtest from nepsy-II-nl [20], measures the ability to receive, process and execute oral instructions of increasing syntactic complexity.	child	*	*	*
*Secondary outcomes*	*Learning conditions test* (Leervoorwaarden test, 24): 70 item questionnaire that measures directly and indirectly learning conditions as concentration, motivation, work rate, task orientation, working according to a plan, persistency, social position in class and relationship with peers and teacher.	teacher	*	*	*
	*Child Behavior Checklist for Ages 6–18 (CBCL/6-18, 25*): 113 item questionnaire designed to measure behaviour problems.	parent	*	*	*
	*Teacher’s Report Form for Ages 6–18 (TRF,* 26): 113 item questionnaire designed to measure behaviour problems.	teacher	*	*	*
	*I-DISC-IV (Diagnostic Interview Schedule for Children,* 27): structured diagnostic telephonic interview, registered via the internet. Measures presence of symptoms and criteria variables as defined by the DSM-IV [14]. Only the domains ADHD, ODD and CD are administered.	parent	*		
	*Social Communication Questionnaire (SCQ, 28*): is a quick screening questionnaire of 40 items for autism spectrum disorders.	parent	*		
	*Kidscreen 27*[29]: 27 item questionnaire that measures quality of life with the dimensions physical well-being, psychological well-being, autonomy & parents, peers & social support and school environment.	child/parent	*	*	*
Intelligence Quotient **	*Shortened version of Wechsler Intelligence Scale for ChildrenIII*[19]*:* subtest Similarities, Block Design, Vocabulary and Information which gives an estimation of the Total IQ	child	*		

T1 = baseline T2 = directly after treatment T3 = follow up after 6 months.

** Intelligence Quotient is only administered if IQ data are not available or when an IQ test has been administered more than two years ago.

### Sample size

For the randomised controlled trial in the first study with two trial arms, 50 children in each trial arm, an effect size of 0.60 can statistically be demonstrated (P < 0.05, 80% power) for comparison of the primary outcome measures of both interventions. This corresponds with an expected clinically relevant difference of 3 points and a standard deviation of 5 on the subtest Digit span from the WISC-III.

For the second study we developed a risk score for the non-responders of the ‘Paying attention in class’ intervention. We expect that 35% (n = 61) of the 175 included children will show no or insufficient result after the 6 month follow up assessment. With 61 “events”, 4 robust true predictors (15 observations per predictor variable) of non-responders can be identified and used for the risk score of non-responders [30].

### Statistical analysis

Multivariate analysis with repeated measures will be conducted, using SPSS Statistics 19 to evaluate training effects on the primary and secondary outcomes.

## Discussion

About 3-5% of the children in the Netherlands is diagnosed with AD(H)D [31]. From earlier research we know that AD(H)D has a strong correlation with working memory problems [32,33] which makes it hard for those children to sufficiently benefit from classroom instructions. Treatment with stimulant medications reduces the behaviour symptoms in children with ADHD but the effects on cognitive functioning are less clear. Given the fact that working memory problems and ADHD are both risk factors for academic failure, it is important to develop and investigate interventions that can be implemented at school. Cogmed is already being applied occasionally by school counseling services but the possible transfer effects on academic performance remain unclear. From practice we know that, after thorough diagnostic assessment, both parents and teachers have an urgent need for specific advice on how to cope with and help the child with specific attention and working memory deficits at school or in daily life. Up till now, clinicians provided such advices based on their theoretical and clinical experience, without a standardized method or practical guide to use. With the first study we hope to gain insight in the short and long-term effects of the working memory – and executive function intervention on neuropsychological functioning, academic performance, behaviour in class, behaviour problems and functioning in every day life. This way we can offer health care professionals, teachers and other educational staff members a more standardized and evidenced based guideline in how to approach and treat children with AD(H)D. As mentioned above, Cogmed is a licensed intervention that entails significant costs for healthcare institutions, health care insurance companies and parents. Our intention is to make our experimental ‘Paying attention in class’ available at a low price, which makes it easily accessible for all schools and school counseling services. Healthcare insurance companies require that treatment programs are proven effective, to which we hope to contribute with this study. The second study should give us insight into the effectiveness of both interventions, taking into account individual characteristics, the level of impairment and the costs (stepped-care approach). If both interventions appear to be effective to improve academic performance, they can be implemented in regular and special education schools as an intervention for students who need extra care.

### Strengths and limitations

Our study has several strengths and limitations. Our first study is randomised and controlled, which is the strongest design to establish efficacy, and the sample size is large. Second, Green and colleagues [13] recommended that academic performance, planning, goal attainment and following rules should be assessed as outcome measures in future studies. Our study includes these recommendations by using tests that have been standardized in Dutch samples. Also we assess the quality of life of the children which hasn’t been done in earlier studies. Finally, in both conditions children receive active treatment and children that do not respond sufficiently to the experimental intervention are offered the Cogmed intervention which had already been proven effective. One limitation of this study is that the interventions from the randomised controlled trial differ on certain aspects, primarily on their content and applicability, which could make it difficult to compare the interventions in terms of efficacy. Another limitation, that has been outlined in recent reviews [34,35], is the use of single tasks to assess the generalization effects of working memory training. It is not clear whether the improved performance reflects change to an underlying ability or reflects practice of a task that strongly resembles trained tasks. Therefore we should be cautious when interpreting results on both near and far transfer effects such as attention or academic performance.

## Competing interests

All authors declare that they have no competing interests.

## Authors’ contributions

MD, AB, AT, AL and RL wrote the current manuscript. MD is the PhD candidate running the study primary in a period of 4 years and will be responsible for collection and analyzing the data. All other authors are supervisors, co investigators and edited the current manuscript. All authors have read and approved the final manuscript.

## Pre-publication history

The pre-publication history for this paper can be accessed here:

http://www.biomedcentral.com/1471-244X/13/23/prepub

## References

[B1] Barkley RA Behavioral inhibition, sustained attention, and executive functions: Constructing a unifying theory of ADHD Psychol Bull 1997 121 1 65 94 9000892 10.1037/0033-2909.121.1.65

[B2] Willcutt EG Doyle AE Nigg JT Faraone SV Pennington BF A meta-analysis of working memory impairments in children with attention-deficit/ hyperactivity disorder: a meta-analytic review Biol Psychiatry 2005 57 1336 1346 10.1016/j.biopsych.2005.02.006 15950006

[B3] Swanson J Baler RD Volkow ND Understanding the effects of stimulant medications on cognition in individuals with attention-deficit hyperactivity disorder: a decade of progress Neuropsychopharmacology 2011 36 207 226 10.1038/npp.2010.160 20881946 PMC3055506

[B4] Bull R Scerif G Executive functioning as a predictor of children’s mathematics ability: inhibition, switching, and working memory Dev Neuropsychol 2001 19 3 273 293 10.1207/S15326942DN1903_3 11758669

[B5] Aronen ET Vuontela V Steenari MR Salmi J Carlson S Working memory, psychiatric symptoms, and academic performance at school Neurobiol Learn Mem 2005 83 33 42 10.1016/j.nlm.2004.06.010 15607686

[B6] Gathercole SE Pickering SJ Working memory deficits in children with low achievements in the national curriculum at 7 years of age Br J Educ Psychol 2000 70 177 194 10.1348/000709900158047 10900777

[B7] Baddeley A The fractionation of working memory Proc Natl Acad Sci 1996 26 13468 13472 8942958 10.1073/pnas.93.24.13468 PMC33632

[B8] Gathercole SE Alloway TP A practical guide for Teachers 2008 London: Sage Publications

[B9] Klingberg T Forssberg H Westerberg H Training of working memory in children with ADHD J Clin Exp Neuropsychol 2002 24 6 781 791 10.1076/jcen.24.6.781.8395 12424652

[B10] Klingberg T Fernell E Olesen P Johnson M Gustafsson P Dahlstrom K Gillberg CG Forssberg H Westerberg H Computerized training of working memory in children with ADHD – a randomized, controlled trial J Am Acad Child Adolesc Psychiatr 2005 44 2 177 186 10.1097/00004583-200502000-00010 15689731

[B11] Holmes J Gathercole SE Dunning DL Adaptive training leads to sustained enhancement of poor working memory in children Dev Sci 2009 12 4 9 15 10.1111/j.1467-7687.2009.00848.x 19635074

[B12] Mezzacappa E Buckner J Working memory training for children with attention problems or hyperactivity: A school-based pilot study Sch Ment Heal 2010 10.1007/s12310-010-9030-9

[B13] Green CT Long DL Green D Iosif A Dixon JF Miller MR Fassbender C Schweitzer JB Will working memory training generalize to improve off-task behavior in children with attention-deficit/hyperactivity disorder Neurotherapeutics 2012 10.1007/s13311-012-0124-y PMC3441930 22752960

[B14] American Psychiatric Association Diagnostic and statistical manual of mental disorders, fourth edition, text revision 2000 Washington, DC: Author

[B15] de Vos T Tempo Test Automatiseren 2010 Amsterdam: Boom test uitgevers

[B16] Geelhoed J Reistma P PI Dictee 1999 Lisse: Swets & Zeitlinger

[B17] Brus BT Voeten MJM Een Minuut Toets 1973 Nijmegen: Berkhout testmateriaal B.V

[B18] Manley T Robertson H Anderson V Nimmo-Smith I Test of Everyday Attention for Children 2004 Enschede: Harcourt Assessment

[B19] Wechsler D Wechsler Intelligence Scale for Children-III-nl 2005 Londen: Harcourt Test Publishers

[B20] Zijlstra HP Kingma A Swaab H Brouwer WH nepsy-II-nl 2010 Enschede Ipskamp

[B21] Wechsler D Naglier JA Wechsler Non Verbal-nl 2008 Amsterdam: Pearson Assessment and Information

[B22] Tjeenk-kalff A Krabbendam L Behavioural Assessment of the Dysexecutive Syndrome for Children BADS-C 2006 Enschede: Drukkerij Printpartners Ipskamp B.V

[B23] Smidts D Huizinga M BRIEF Executieve Functies Gedragsvragenlijst 2009 Amsterdam: Hogrefe Uitgevers B.V

[B24] Scholte EM van der Ploeg JD Leervoorwaardentest (LVT) 2011 Houten: Bohn Stafleu van Loghum

[B25] Verhulst FC van der Ende J Koot HM Handleiding Child Behaviour Checklist 4–18 1996 Rotterdam: Afdeling Kinder –en Jeugdpsychiatrie, Sophia Kinderziekenhuis/Academisch Ziekenhuis Rotterdam/Erasmus Universiteit van Rotterdam

[B26] Verhulst FC van der Ende J Koot HM Handleiding Teacher’s Report Form 1997 Rotterdam: Afdeling Kinder –en Jeugdpsychiatrie, Sophia Kinderziekenhuis/Academisch Ziekenhuis Rotterdam/Erasmus Universiteit van Rotterdam

[B27] Steenhuis MP Serra M Minderaa RB Hartman CA An internet version of the diagnostic interview schedule for children (DISC-IV): correspondence of the ADHD section with the paper-and-pencil version Psychol Assess 2009 21 2 231 234 19485678 10.1037/a0015925

[B28] Warreyn P Raymaekers R Roeyers H SCQ: Vragenlijst Sociale Communicatie 2004 Destelbergen: Vormingsdienst SIG vzw

[B29] Ravens-Sieberer U Auquier P Erhart M Gosch A Rajmil L Bruil J Power M Dür W Cloetta B Czemy L Mazur J Czimbalmos A Tountas Y Hagquist C Kilroe J KIDSCREEN Group The Kidscreen-27 quality of life measure for children and adolescent: psychometric results from a cross-cultural survey in 13 European countries Qual Life Res 2007 16 8 1347 1356 10.1007/s11136-007-9240-2 17668292

[B30] Babyak MA What you see may not be what you get: A brief, nontechnical introduction to overfitting in regression-type models Psychosom Med 2004 66 411 421 10.1097/01.psy.0000127692.23278.a9 15184705

[B31] Landelijke Stuurgroep Multidisciplinaire richtlijnontwikkeling in de GGZ Richtlijn voor de diagnostiek en behandeling van ADHD bij kinderen en jeugdigen 2005 Utrecht: Trimbos-Instituut

[B32] Kofler MJ Rapport MD Bolden J Sarver DE Raiker JS ADHD and working memory: the impact of central executive deficits and exceeding storage/rehearsal capacity on observed inattentive behavior J Abnorm Child Psychol 2010 38 149 161 10.1007/s10802-009-9357-6 19787447

[B33] Rommelse NNJ Van der Stigchel S Witlox J Geldof C Deijen JB Theeuwes J Oosterlaan J Sergeant JA Deficits in visuo-spatial working memory, inhibition and oculomotor control in boys with ADHD and their non-affected brothers J Neural Transm 2008 115 249 260 10.1007/s00702-007-0865-7 18253811

[B34] Shipstead Z Redick TS Engle RW Is working memory training effective Psychol Bull 2012 10.1037/a0027473 22409508

[B35] Morrison AB Chein JM Does working memory training work? The promise and challenges of enhancing cognition by training working memory Pscyhon Bull Rev 2011 18 46 60 10.3758/s13423-010-0034-0 21327348

